# Phase I Radiation Dose-Escalation Study to Investigate the Dose-Limiting Toxicity of Concurrent Intra-Arterial Chemotherapy for Unresectable Hepatocellular Carcinoma

**DOI:** 10.3390/cancers12061612

**Published:** 2020-06-18

**Authors:** Yeona Cho, Jun Won Kim, Ja Kyung Kim, Kwan Sik Lee, Jung Il Lee, Hyun Woong Lee, Kwang-Hun Lee, Seung-Moon Joo, Jin Hong Lim, Ik Jae Lee

**Affiliations:** 1 Department of Radiation Oncology, Gangnam Severance Hospital, Yonsei University College of Medicine, Seoul 06273, Korea; iamyona@yuhs.ac (Y.C.); junwon@yuhs.ac (J.W.K.); 2 Department of Internal Medicine, Yongin Severance Hospital, Yonsei University College of Medicine, Yongin 16995, Korea; CECILIAK@yuhs.ac; 3 Department of Internal Medicine, Gangnam Severance Hospital, Yonsei University College of Medicine, Seoul 06273, Korea; LEEKS519@yuhs.ac (K.S.L.); MDFLORENCE@yuhs.ac (J.I.L.); LHWDOC@yuhs.ac (H.W.L.); 4 Department of Radiology, Gangnam Severance Hospital, Yonsei University College of Medicine, Seoul 06273, Korea; DOCTORLKH@yuhs.ac (K.-H.L.); HUCHI79@yuhs.ac (S.-M.J.); 5 Department of Surgery, Gangnam Severance Hospital, Yonsei University College of Medicine, Seoul 06273, Korea

**Keywords:** Hepatocellular carcinoma, radiotherapy, chemoradiotherapy, toxicity

## Abstract

Concurrent intra-arterial chemotherapy and radiotherapy (iA-CCRT) can increase the response rate in hepatocellular carcinoma (HCC), but may cause a higher toxicity. We conducted this Phase I study to investigate the dose-limiting toxicity of iA-CCRT for HCC. In total, 52.5 Gy in 25 fractions was prescribed as planning target volume (PTV) 1 at dose level 1. The dose escalation was 0.2 Gy per fraction and up to 2.5 Gy, with 62.5 Gy at level 3. Concurrent intra-arterial 5-fluorouracil was administered during the first and fifth weeks of radiotherapy (RT). Toxicities were graded using the Common Toxicity Criteria for Adverse Events, version 4.0. Results: Seventeen patients with HCC were analyzed: four at dose level 1, 6 at level 2, and 7 at level 3. The mean irradiated dose administered to the uninvolved liver at each dose level was 21.3, 21.6, and 18.2 Gy, respectively. There was no grade ≥3 gastrointestinal toxicity; two patients experienced grade 3 hyperbilirubinemia. All patients had Child-Pugh class A disease, but 3 patients developed class B disease after iA-CCRT. During a median follow-up of 13 months, the median progression-free survival (PFS) and overall survival (OS) were 10 and 22 months, respectively. Patients treated at dose level 3 showed improved PFS and OS. Conclusions: Radiation dose escalation of iA-CCRT did not cause any significant toxicities in patients with advanced HCC. Further large-scale studies with long-term follow-up are needed to determine the efficacy and feasibility of higher doses of iA-CCRT.

## 1. Introduction

Hepatocellular carcinoma (HCC) is the fifth most common malignancy worldwide [1]. Despite recent advances in diagnostic and therapeutic techniques, HCC prognosis is poor, especially in locally advanced cases. Resection is the most effective method for treating HCC; the 5-year survival rate after the resection of early HCC is 50–70% [2]. However, resection has a limited role in treating advanced HCC; most patients with advanced HCC are not appropriate candidates for surgery at the time of diagnosis, owing to their poor liver function, wide intrahepatic distribution, vascular invasion, and comorbidities [3].

Radiofrequency ablation is an alternative local treatment, but the procedure can be complicated when the tumor is adjacent to the gallbladder, major vessels, or the diaphragm [4]. Although transarterial chemoembolization (TACE) has survival benefits compared to systemic treatment [5,6], TACE alone frequently results in incomplete tumor necrosis and requires repeated TACE, eventually becoming less effective [7].

Radiotherapy (RT) is an alternative treatment for locally advanced HCC, particularly Barcelona clinic liver cancer (BCLC) stage C HCC [8,9,10], for which there is no specific treatment except sorafenib. RT has been attempted for intrahepatic tumors since the 1970s; the entire liver was irradiated, resulting in unsatisfactory responses with insufficient radiation doses [11]. However, recent advances in RT technology, such as intensity-modulated RT (IMRT) and image-guided RT (IGRT), have made high-dose localized RT possible, without significant toxicities [12,13,14,15]. Hence, patients who cannot undergo resection may be treated with combined local RT and intra-arterial chemotherapy as a new treatment strategy [16,17,18]. RT administration has especially increased in cases where there is no satisfactory response to TACE or when toxicities increase with repeated TACE usage [19,20,21,22,23]. A recent randomized clinical trial also reported that TACE along with RT improved survival compared to systemic treatment in patients with macroscopic vascular invasion [24].

The concurrent use of intra-arterial chemotherapy and RT (iA-CCRT) can increase the response rate, but may cause a higher toxicity than that caused by the single use of each treatment [25,26]. IMRT and IGRT improved the oncologic outcome of HCC [27]. Furthermore, concurrently administering 45 Gy RT and 5-fluorouracil (FU) chemotherapy into the hepatic artery yielded excellent results in terms of increasing RT’s effectiveness and reducing intrahepatic metastasis [17,28]. Although RT has a dose-response relationship for treating HCC [29], no study has evaluated the optimal radiation dose for combined treatments.

Therefore, this Phase I clinical study investigated the dose-limiting toxicity of RT and 5-FU chemotherapy by increasing the radiation dose in patients with unresectable primary HCC.

## 2. Results

### 2.1. Patient Characteristics

Seventeen patients were enrolled in this study between August 2015 and November 2018; four patients were treated at dose level 1, 6 at dose level 2, and 7 at dose level 3. Two patients were excluded: one patient had distant metastasis at enrollment and the other withdrew from this study.

The patients and tumor characteristics are shown in Table 1. The median patient age was 63 years (range, 33–80 years). More than 70% of patients had hepatitis B or C virus infections, resulting in pre-RT liver cirrhosis in 76.5% of patients. Among the four patients without either hepatitis B or C infections, two patients presented with underlying alcoholic liver cirrhosis and the third one with fatty liver disease. The fourth patient had no underlying liver disease except for HCC at the time of diagnosis. The median levels of α-fetoprotein (AFP) and protein induced by vitamin K absence or antagonist-II (PIVKA-II) were 45 ng/mL and 381.4 mIU/mL, respectively. All patients had a good liver function, as demonstrated by Child-Pugh class A. Only two patients showed moderate thrombocytopenia.

Table 2 demonstrates the dosimetric parameters based on the radiation dose levels. The median planning target volume (PTV) 1 volumes at levels 1, 2, and 3 were 397.6, 489.9, and 354.8 cc, respectively. The corresponding median volumes of the uninvolved liver were 1018, 1138, and 1176 cc, respectively. The corresponding mean irradiated doses administered to the uninvolved liver were 21.3, 21.6, and 18.2 Gy, respectively.

### 2.2. Toxicities

The treatment protocol in this study was well-tolerated. No patient experienced dose-limiting toxicities (DLTs). Treatment-related toxicities are shown in Table 3. Only one patient experienced grade 2 gastrointestinal (GI) toxicity (nausea/vomiting), and there were no grade ≥3 GI toxicities. Most toxicities that affected the liver function were grade 1 or 2 toxicities. Grade 1 or 2 alkaline phosphatase (ALP) elevation not associated with ascites or hepatomegaly was observed in 88.2% of patients. However, two patients experienced grade 3 hyperbilirubinemia.

**Figure 1:**
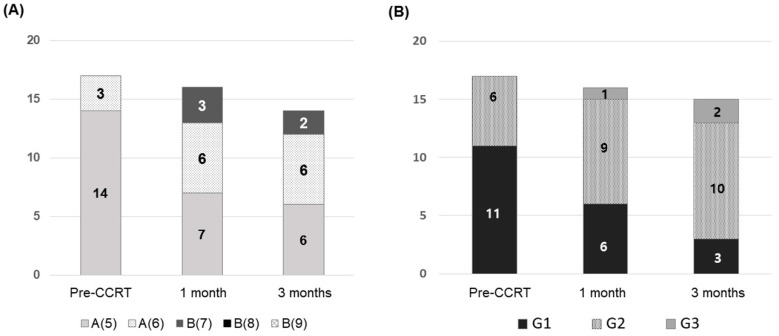
Changes of the liver function: (**A**) Child-Pugh score and (**B**) albumin-bilirubin grade before and after concurrent intra-arterial chemotherapy and radiotherapy (iA-CCRT).

Before iA-CCRT, all patients had Child-Pugh class A disease with 5–6 points, but the proportion of patients with class B disease increased over time after iA-CCRT (Figure 1A); three patients had class B disease within 3 months after iA-CCRT. Of the two patients who showed an increase of ≥2 points in the Child-Pugh score within 3 months after iA-CCRT completion, one had intrahepatic failure despite iA-CCRT, resulting in non-classic radiation-induced liver disease (RILD). Figure 1B shows the proportion of each albumin-bilirubin (ALBI) grade before and after iA-CCRT. No patient had grade 3 ALBI, but after 3 months, two patients had grade 3 ALBI. All of these patients died of disease progression.

Almost all patients showed a grade 1 increase in the international normalized ratio (INR); most of these had an increased INR at iA-CCRT initiation. Two patients showed grade 3 neutropenia and two had grade 3 thrombocytopenia. None of the patients experienced grade ≥3 general weakness.

### 2.3. Treatment Outcomes

**Figure 2:**
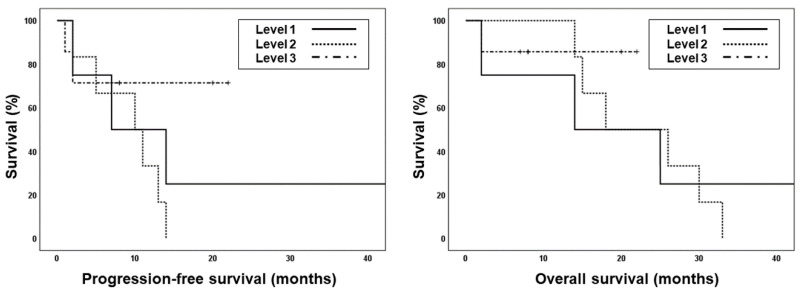
Kaplan–Meir survival curves showing progression-free survival (PFS, dashed line) and overall survival (OS, solid line) of each dose level.

**Figure 3:**
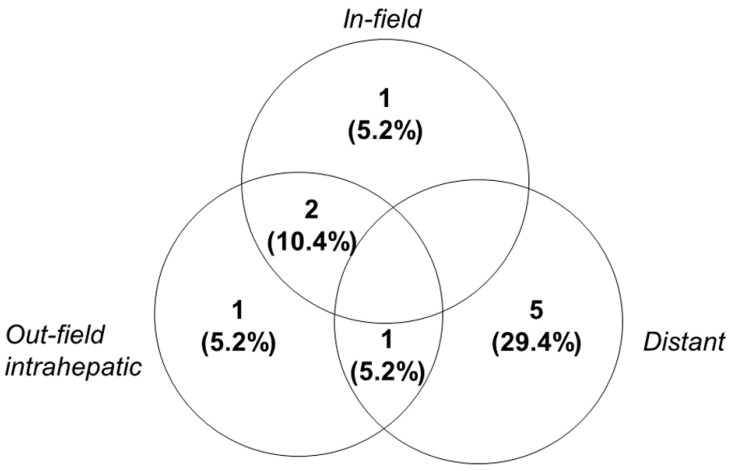
Sites of first disease recurrence. In-field, within the high-dose irradiated volume (i.e., 80% isodose line of the prescribed dose in the planning target volume; out-field, outside the high-dose irradiated volume).

During the median follow-up of 13 months, nine patients experienced disease progression and 10 died. Among the 10 patients who died, one died of esophageal varix bleeding 2 months after iA-CCRT and one died of aspiration pneumonia 32 months after iA-CCRT; eight patients experienced cancer-specific death (47.1%). The median progression-free survival (PFS) and overall survival (OS) were 10 months and 22 months, respectively (Figure S2). The median follow-up durations for patients at dose levels 1, 2, and 3 were 13, 18, and 8 months, respectively. Patients treated at dose level 3 showed better PFS and OS than those at levels 1 or 2 (median PFS: 7 and 10 months vs. not-reached; median OS: 14 and 18 months vs. not-reached, Figure 2); however, the results were not significant. The most common pattern of failure was distant metastases only (29.4%, Figure 3); four patients developed lung metastases and one had mediastinal lymph node metastases.

Within 3 months after treatment, four patients showed a complete response (CR) and 10 showed a partial response (PR), resulting in an overall response rate of 82.4%. The overall response rate was the highest at dose level 3, i.e., 85.7% compared to that at level 1 (75%) and level 2 (83.3%).

## 3. Discussion

We evaluated the dose-limiting toxicity of RT and 5-FU chemotherapy by increasing the radiation dose in patients with unresectable primary HCC, and there was no significant treatment-related GI toxicity. Only two patients showed an increase of ≥2 points in the Child-Pugh score; however, these might be associated with disease progression.

There are no clear guidelines regarding the optimal dose in HCC radiotherapy. However, a higher radiation dose was significantly associated with HCC response [29]. In addition, patients who received additional RT after incomplete TACE showed an improvement in local failure-free survival and PFS without significant toxicities in patients who received RT with ≥72 Gy (biologically effective dose (BED), α/β = 10) [19]. An iA-CCRT for BCLC stage C HCC also improved the survival of patients treated with ≥72 Gy (BED, α/β = 10); the median survival was 20.7 months [30]. They focused on the simultaneous integrated boost technique using IMRT. In the current study, a higher RT dose at level 3 (BED, 78.13 Gy with α/β = 10) was associated with prolonged PFS and OS. Nevertheless, Phase II and III studies using a dose level ≥ 3 could provide more information regarding the optimal RT dose with a low toxicity while improving treatment outcomes.

Several Phase I dose-escalation studies have evaluated the optimal dose of stereotactic body radiotherapy (SBRT) for HCC. No DLT was observed within 3 months after the SBRT of 54 Gy in six fractions for HCC or intrahepatic cholangiocarcinoma [31]. A higher SBRT dose (60 Gy in four fractions) was also safe [32,33]. Although SBRT showed a favorable response in patients with HCC without significant toxicities, most patients have multiple and large tumors, as well as vascular invasion. Moreover, the organs at risk (OARs) need to be considered more strictly for patients treated with SBRT. Therefore, only a few patients with HCC are candidates for SBRT, and the toxicities of high-dose RT with conventional fractionation should be evaluated.

Most studies evaluating high-dose RT are retrospective and the RT regimen is heterogeneous, making it difficult to determine the toxicity profile. In the current study, dose escalation was gradually performed according to the protocols, and we prospectively performed a toxicity evaluation. Although most toxicities were grade 1 or 2, several patients showed grade 3 toxicity associated with the liver function within 3 months of treatment completion. Although these might be iA-CCRT-related toxicities, they might also be due to tumor progression or worsening of the underlying liver disease. It was challenging to make a clear distinction because most patients eventually died of distant metastasis or hepatic failure due to disease progression.

RILD is among the most severe complications that can occur after liver-direct RT. Patients in this study were more likely to have non-classic RILD because most of them had underlying liver diseases, such as viral infection or liver cirrhosis. None of the patients had classic RILD, but one patient showed an increase of two points in the Child-Pugh score without disease progression within 3 months of treatment. A recent multicenter, retrospective study in the Korean population showed that approximately 20% of the population had non-classic RILD, and a normal liver volume was the most predictive dosimetric parameter of non-classic RILD [34]. Patients whose uninvolved liver volume was ≥800 cc were included in the current study; only one patient had non-classic RILD, despite dose escalation. As normal tissue toxicity is greatly influenced by the fraction size, fractionated conformal RT should be used for patients with Child-Pugh class B disease to minimize toxicity [35]. The mechanisms of RILD development remain largely unknown, and there is no effective therapy to stop RILD progression. Conservative care with anticoagulant therapy is mainly performed with warfarin; recently, glutathione, selenium/vitamin E, or defibrotide (a fibrinolytic agent) was used, but no studies have evaluated the clinical effects [36]. Therefore, RILD prevention is crucial, and the underlying mechanism should be investigated. Additionally, it is necessary to comply with dose-based recommendation criteria considering the liver function and tumor size.

ALBI grade—utilizing albumin and bilirubin—is a new model for predicting the liver function. ALBI is associated with disease progression and the survival of patients with HCC [37,38]. All patients in this study with ALBI grade 3 at 3 months died of disease progression, suggesting that ALBI may be a prognostic factor in patients receiving iA-CCRT. The ALBI grade is more objective and predictable in those treated with RT, especially those with minimal liver dysfunction [39]. Moreover, ALBI is simple, objective, and clinically feasible in comparison to other parameters for evaluating the liver function [40,41]. Therefore, it is essential to find suitable candidates for iA-CCRT, considering the various parameters of the liver function.

In the present study, most patients did not experience severe radiation toxicity, but because their tumor sizes were relatively large, concerns about the deterioration of the liver function cannot be ignored. Byun et al. attempted dose-escalation for large tumors and reported a median PTV2 of 1111 cc, which is larger than the one observed in the present study (784 cc); other studies have also reported that SBRT or IMRT can be relatively safely used, even for large tumors [30,42,43]. However, proton therapy or heavy particle therapy may be advantageous to preserve the OAR, particularly liver tissue. Since the Bragg peak properties of proton therapy and heavy particle therapy allow for improved conformality of the treatment field, large tumor volumes can be irradiated with high doses without significant dose exposure to surrounding normal liver tissue [44,45]. Our institution is planning to introduce Carbon Ion Therapy in the following year, which we believe will play a crucial role in the treatment of unresectable HCC. Several studies will be conducted in this regard.

This study had several limitations. As we only included a small number of patients, most results were not statistically confirmed. Moreover, the follow-up period for patients treated at dose level 3 was shorter than that for the other two groups; this may have influenced the favorable outcome of these patients. Therefore, the efficacy of high-dose RT requires longer follow-up with a larger number of patients. Furthermore, patients included in this study had different disease statuses, and patients who had previously received other local treatments were also included. Therefore, these results should be interpreted with caution. Nevertheless, to the best of our knowledge, the current study is the first prospective study to evaluate the DLTs of iA-CCRT.

## 4. Materials and Methods

### 4.1. Patient Eligibility

This prospective single-institution Phase I study was approved by the Institutional Review Board (IRB, protocol number: 3-2015-0102) in 2015 and Clinical Research Information Service (the clinical research registry system of Korea). Patients with unresectable HCC who consented to being involved in this study between August 2015 and November 2018 were included. The eligibility criteria were as follows: (1) HCC not treatable with surgery or other local treatment; (2) age ≥20 years; (3) Eastern Cooperative Oncology Group (ECOG) performance status score of 0–2; (4) Child-Pugh score of 5–7; (5) uninvolved liver volume >800 cc; (6) sufficient distance (≥0.5 cm) of the tumor(s) from adjacent OARs, including the duodenum, stomach, and small bowel; (7) an adequate liver function (aspartate aminotransferase [AST]/alanine aminotransferase [ALT] <5 times the upper limit of the normal value, total bilirubin <3 mg/dL, albumin >2.5 g/dL, a normal prothrombin time (PT), and a partial thromboplastin time (PTT)); (8) an adequate renal function (serum creatinine < 1.8 mg/dL or clearance of creatinine >50 mL/min); (9) a reserved bone marrow function (absolute neutrophil count ≥1500/mm^3^, platelet count ≥50,000/mm^3^, and hemoglobin >9 g/dL); and (10) a measurable and assessable lesion when using computed tomography (CT). Patients were excluded if they had previously received RT in the abdominal area, had distant metastasis, and did not provide informed consent.

For HCC diagnosis, a biopsy was not required if the tumors were enhanced on two imaging modalities and the AFP level was high in a patient with a known background of liver disease [18]. The treatment for HCC was determined by a multidisciplinary team of radiologists, hepatic surgeons, transplant surgeons, radiation oncologists, and medical oncologists. Usually, in the case of a resectable tumor, surgery was considered first, and further treatment was decided on the basis of the size, number, or location of the tumor; the presence of lymph node metastasis; or portal vein thrombosis. iA-CCRT was usually considered as a treatment modality for unresectable tumors when other treatment options were not suitable due to the size, number, or location of the tumor; portal vein thrombosis; LN metastasis; or if previous therapies failed.

### 4.2. Simulation and Radiotherapy Planning

Patients underwent respiratory training prior to CT simulation to ensure regular breathing during simulation and radiation treatment. After 4 h of fasting on the day of the simulation, patients underwent CT simulation for RT planning. We also used a body fixation device and an abdominal compression device. Moreover, four-dimensional CT was performed to evaluate the movement of tumors and OARs and to determine internal margins.

We used simultaneous integrated boost techniques along with IMRT. The internal target volume (ITV) was determined by considering the gross tumor volume during all respiratory cycles on 4D-CT images. The ITV was considered the PTV1. The clinical target volume (CTV) was the PTV1 with a uniform 5-mm margin. PTV2 was the CTV with a 5-mm radial margin and 7-mm craniocaudal margin.

PTV1 was included in the 90% isodose curve of the prescribed dose. At dose level 1, 52.5 Gy in 25 fractions was prescribed for PTV1 with fractional doses of 2.1 Gy (Table S1). The dose escalation for PTV1 was 0.2 Gy per level up to 2.5 Gy, with 62.5 Gy at level 3. In total, 45 Gy in 25 fractions was prescribed for PTV2 at level 1. The fractional dose for PTV2 was increased by 0.2 Gy at level 2, and 50 Gy was prescribed. However, at level 3, further dose escalation was not performed for PTV2, limiting the total dose to 50 Gy for PTV2. The PTV was included in the 90% isodose curve of the prescribed dose, and doses were customized to satisfy the normal organ dose constraints (Table S2). Over 90% of the dose prescribed for PTV1 was delivered to PTV1. If PTV1 was close to the gastrointestinal structures, including the stomach and duodenum, the minimum distance-to-target was set at 5 mm [46]. RT was delivered using a 6 MV linear accelerator (Versa HD, Elekta, Stockholm, Sweden) or Helical TomoTherapy^®^ (Accuray Inc., Sunnyvale, CA, USA). The radiation oncologists from our institution have many years of experience in administering IMRT for HCC, as described previously [27,47,48].

### 4.3. Intra-Arterial Chemotherapy

During RT, concurrent 5-FU was continuously injected into the hepatic artery using a percutaneous hepatic artery catheter. The intra-arterial 5-FU dose was 500 mg/day, administered five times a week during the first and fifth weeks of RT. After RT completion, cisplatin (60 mg/m^2^) was added to 5-FU on the second and third days of each chemotherapy cycle. Depending on the response, an additional 3–12 cycles of chemotherapy were administered.

### 4.4. Dose-Limiting Toxicity

Toxicities were graded using the National Cancer Institute Common Toxicity Criteria for Adverse Events (CTCAE), version 4.0. Classic RILD was defined as an increase in anicteric ascites, hepatomegaly, and an elevation in ALP (two times higher than the baseline ALP level) in the absence of disease progression. Ascites could be detected on abdominal CT, and cytology was performed to confirm the presence of cancer cells in the ascites. Moreover, there should have been no evidence of a deterioration in the liver function or intraperitoneal seeding metastasis. Non-classic RILD was defined as a dysregulated liver function, with remarkably elevated serum transaminase levels (>5 times the upper limit of the normal level) or worsening of the Child-Pugh score by ≥2 points with underlying liver disease, such as viral infection or liver cirrhosis [34].

DLT was defined as (1) any CTCAE grade ≥4 GI toxicity; (2) persistent grade 3 GI toxicity, despite proper management; (3) RILD requiring treatment within 3 months after RT completion; (4) interruption of RT for >2 weeks; or (5) incomplete RT.

At least three patients treated at each level were enrolled and received treatment at the next level if DLTs did not occur within 3 months after RT completion. When one or two patients experienced DLTs, at least three additional patients were added to that dose level (Figure S1). If sufficient follow-up was not achieved, the current level was maintained and patients did not receive treatment at the next level.

### 4.5. Follow-Up and Analysis

Patients were assessed 1, 3, 6, and 12 months after completing RT to evaluate treatment responses and toxicities. At the 1- and 3-month follow-ups, dynamic liver CT was performed. Magnetic resonance imaging or endoscopic gastroduodenoscopy was performed when indicated. Laboratory data, including common blood test measurements, liver enzymes, and tumor markers, were also evaluated. The Child-Pugh score was calculated using five variables, including bilirubin, albumin, and prothrombin levels; the ascites status; and the degree of encephalopathy. The ALBI score was calculated [38] as follows:

ALBI score = (log10 bilirubin [μmol/L] × 0.66) + (albumin [g/L] × −0.0852).

ALBI score ≤−2.60 indicated grade 1 ALBI; >−2.60 to ≤−1.39 indicated grade 2 ALBI; and >−1.39 indicated grade 3 ALBI.

The tumor response was assessed using Modified Response Evaluation and Criteria in Solid Tumors (mRECIST). At 1 and 3 months after RT, the response rates were analyzed: responders included those who showed a CR and partial response PR, and non-responders included those with stable disease (SD) and progressive disease (PD). In-field recurrence was defined as recurrence within the high-dose region (>80% isodose volume), demonstrated by new enhancement or PD on RECIST.

The progression-free survival (PFS) and overall survival (OS) rates were evaluated by using the Kaplan–Meier method. All analyses were performed using IBM SPSS, version 24.0 (SPSS, Chicago, IL, USA).

## 5. Conclusions

Radiation dose escalation combined with intra-arterial chemotherapy showed no significant RT-induced toxicities in patients with advanced HCC. However, additional Phase II and III studies including large populations with long-term follow-up could determine the efficacy and feasibility of a higher dose (≥78 Gy, BED) of iA-CCRT.

## Figures and Tables

**Table 1:** Patients and tumor characteristics.

Characteristics	Median	(Range)	No. of Patients (*n* = 17)	(%)
Age	63	(33–80)		
Sex				
Male			15	(88.2)
Female			2	(11.8)
ECOG PS				
0			8	(47.1)
1			9	(52.9)
Underlying liver disease				
HBV			12	(70.6)
HCV			1	(5.9)
Without viral infections			4	(23.5)
Underlying liver cirrhosis				
No			4	23.5
Yes			13	76.5
AFP (ng/mL)	45	(2.2–38,300)		
>9 ng/mL			12	70.6
PIVKA-II (mIU/mL)	381.4	(23–185,072)		
>35 mIU/mL			13	76.5
Child-Pugh class				
A5			14	82.4
A6			3	17.6
Platelet count	163 k	(55–408 k)	5	29.4
Mild thrombocytopenia (75–150 k/uL)			3	15.8
Moderate thrombocytopenia (50–75 k/μL)			2	11.8
UICC stage				
T2			2	11.8
T3			8	47.1
T4			7	41.2
N0			16	94.1
N1			1	5.9
Primary tumor size (cm)	8	(2.6–16)		
Number of tumor(s)				
1			8	47.1
2–4			7	41.2
≥5			2	11.8
Involved site				
Right Lobe			11	64.7
Left Lobe			2	11.8
Both Lobes			4	23.5
Vascular invasion				
No			3	17.6
Yes			14	82.4
Previous treatment				
None			13	76.5
TACE			4	23.5
TACI			1	5.9
RFA			1	5.9

Abbreviations: ECOG PS, Eastern Cooperative Oncology Group performance status; AFP, alpha fetoprotein; PIVKA-II, protein induced by vitamin K absence or antagonist II; RT, radiotherapy; TACE, transcatheter arterial chemoembolization; TACI, transcatheter arterial chemoinfusion; RFA, radiofrequency ablation. Most patients had locally advanced disease (88.2% had ≥T3 disease); one patient had lymph node metastasis. The median primary tumor size was 8 cm, and 47.4% of patients had multiple intrahepatic tumors; 21.1% of tumors involved both lobes. Most patients (73.7%) showed vascular invasion. Thirteen patients (68.4%) received no treatment before concurrent intra-arterial chemotherapy and radiotherapy (iA-CCRT).

**Table 2:** Dosimetric parameters based on radiation dose levels.

	Level 1		Level 2		Level 3		Total	
	(*n* = 4)		(*n* = 6)		(*n* = 7)		(*n* = 17)	
Parameters	Median	(Range)	Median	(Range)	Median	(Range)	Median	(Range)
PTV1 (cc)	398	(277–467)	490	(69–2086)	355	(260–909)	398	(69–2086)
PTV2 (cc)	819	(561–2066)	717	(209–2814)	758	(525–1634)	784	(209–2814)
Uninvolved liver volume (cc)	1018	(876–1643)	1138	(814–1393)	1176	(855–1511)	1122	(814–1643)
Mean dose of whole liver (Gy)	30.4	(20.5–42.1)	28.1	(18.8–38.2)	30.1	(17.7–39.6)	30.4	(18.8–42.1)
Mean dose of uninvolved liver (Gy)	21.3	(15.35–27.4)	21.6	(19.2–25.7)	18.2	(11.5–24.4)	20.4	(11.5–27.4)
Maximum dose of stomach (Gy)	42.6	(20.3–55.4)	27.9	(15.1–54.0)	51.2	(26.8–56.3)	40.9	(15.1–56.3)
Maximum dose of duodenum (Gy)	40.0	(21.4–52.2)	37.8	(2.1–54.6)	48.5	(15.1–54.1)	40	(2.1–54.6)
Maximum dose of spinal cord (Gy)	29.8	(26.8–35.2)	25.7	(18.7–37.4)	36.8	(24.7–44.0)	30.5	(18.7–44.0)
Mean dose of right kidney (Gy)	5.6	(2.5–19.4)	6.4	(1.1–20.4)	2.8	(1.3–17.5)	6.4	(1.1–20.4)
Mean dose of left kidney (Gy)	4.6	(0.8–16.9)	3.2	(0.6–6.9)	2.4	(0.6–7.5)	3.2	(0.6–16.9)

Abbreviations: PTV, planning target volume.

**Table 3:** Treatment-related toxicities within 3 months after concurrent chemoradiotherapy.

		Level 1 (*n* = 4)			Level 2 (*n* = 6)			Level 3 (*n* = 7)			Total (*n* = 17)		
		N (%)			N (%)			N (%)			N (%)		
Toxicities		G1	G2	G3	G1	G2	G3	G1	G2	G3	G1	G2	G3
GI toxicity	Nausea	0	0	0	0	0	0	0	1 (14.3)	0	0	1 (5.9)	0
	Vomiting	0	0	0	0	0	0	0	0	0	0	0	0
	Pain	1 (25)	0	0	1 (16.7)	1 (16.7)	0	1 (14.3)	0	0	3 (17.6)	1 (5.9)	0
Liver function	AST	2 (50)	1 (25)	0	5 (83.3)	0	0	6 (85.7)	0	0	13 (76.5)	1 (5.9)	0
	ALT	2 (50)	0	0	0	0	0	4 (57.1)	0	0	6 (35.3)	0	0
	Albumin	3 (75)	1 (25)	0	4 (66.7)	1 (16.7)	0	4 (57.1)	1 (14.3)	0	11 (64.7)	3 (17.6)	0
	Bilirubin	0	0	1 (25)	0	2 (33.3)	0	0	0	1 (14.3)	0	2 (11.8)	2 (11.8)
	INR	4 (100)	0	0	6 (100)	0	0	5 (71.4)	0	0	15 (88.2)	0	0
	ALP	2 (50)	1 (25)	0	5 (83.3)	0	0	4 (57.1)	3 (42.9)	0	11 (64.7)	4 (23.5)	0
Hematologic	Hemoglobin	2 (50)	2 (50)	0	2 (33.3)	0	0	3 (42.9)	0	1 (14.3)	7 (41.2)	2 (11.8)	1 (5.9)
	WBC	1 (25)	2 (50)	0	2 (33.3)	0	0	2 (28.6)	3 (42.9)	0	5 (29.4)	5 (29.4)	0
	ANC	2 (50)	0	1 (25)	1 (16.7)	1 (16.7)	0	0	4 (57.1)	1 (14.3)	3 (17.6)	5 (29.4)	2 (11.8)
	Platelet	2 (50)	1 (25)	1 (25)	0	1 (16.7)	1 (16.7)	0	2 (28.6)	0	2 (11.8)	4 (23.5)	2 (11.8)
Other	General weakness	0	1 (25)	0	3 (50)	0	0	0	1 (14.3)	0	3 (17.6)	2 (11.8)	0

Abbreviations: GI, gastrointestinal; AST, aspartate aminotransferase; ALT, alanine aminotransferase; INR, international normalized ratio; ALP, alkaline phosphatase; WBC, white blood cell; ANC, absolute neutrophil count.

## Supplementary Materials

The following are available online at https://www.mdpi.com/2072-6694/12/6/1612/s1, Figure S1: Protocol scheme for the current Phase I study, Figure S2: Progression-free survival and overall survival of all patients (*n* = 17), Table S1: Dose escalation schedule, Table S2: Normal tissue radiation dose constraints.

## Abbreviation

AFP	α-fetoprotein
ALP	Alkaline phosphatase
BCLC	Barcelona clinic liver cancer
CR	Complete response
CT	Computed tomography
CTCAE	Common Toxicity Criteria for Adverse Events
CTV	Clinical target volume
DLT	Dose-limiting toxicity
ECOG	Eastern Cooperative Oncology Group
HCC	Hepatocellular carcinoma
IGRT	Image-guided radiotherapy
IMRT	Intensity-modulated radiotherapy
INR	International normalized ratio
IRB	Institutional Review Board
ITV	Internal target volume
OAR	Organs at risk
OS	Overall survival
PD	Progressive disease
PFS	Progression-free survival
PR	Partial response
PTV	Planning target volume
RILD	Radiation-induced liver disease
SBRT	Stereotactic body radiotherapy
SD	Stable disease
TACE	Transarterial chemoembolization
