# Supplementary material for: Phase I Radiation Dose-Escalation Study to Investigate the Dose-Limiting Toxicity of Concurrent Intra-Arterial Chemotherapy for Unresectable Hepatocellular Carcinoma
Source: Cancers (Basel). 2020 Jun 18;12(6):1612. doi: 10.3390/cancers12061612 (PMC7352219; doi:10.3390/cancers12061612)
Supplement: Supplementary file 1 [file cancers-12-01612-s001.pdf]

Supplementary Materials: Phase I Radiation Dose-Escalation Study to Investigate the Dose-Limiting Toxicity of Concurrent Intra-Arterial Chemotherapy for Unresectable Hepatocellular Carcinoma

Yeona Cho, Jun Won Kim, Ja Kyung Kim, Kwan Sik Lee, Jung Il Lee, Hyun Woong Lee, Kwang-Hun Lee, Seung-Moon Joo, Jin Hong Lim and Ik Jae Lee

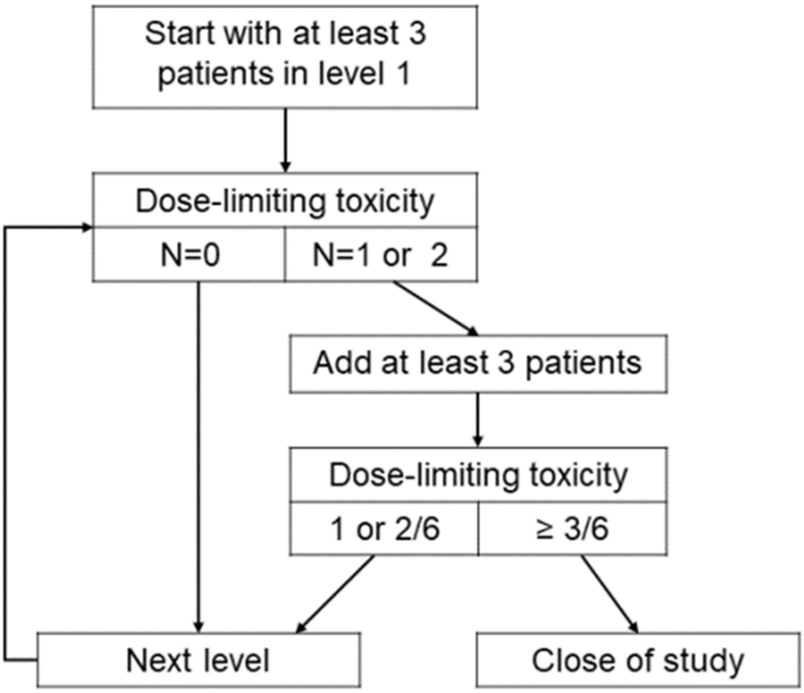

Figure S1. Protocol scheme for the current Phase I study.

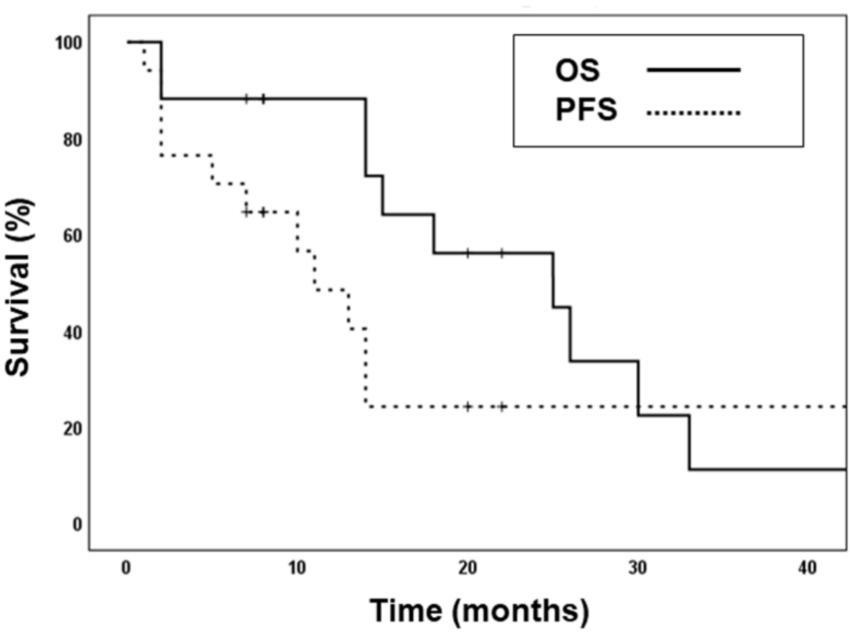

Figure S2. Progression-free survival and overall survival of all patients (n = 17).

Table 1. Dose escalation schedule.

Dose level	PTV1 (Gy)	BED10 (Gy)	BED3 (Gy)	PTV2 (Gy)	Fractions	No. of patients
1	2.1	63.53	89.25	1.8	25	4
2	2.3	70.72	101.58	2.0	25	6
3	2.5	78.13	114.58	2.0	25	7

Abbreviations: PTV, planning target volume; BED10, biologically effective dose with α/β ratio = 10; BED3, biologically effective dose with α/β ratio = 3.

Table S2. Normal tissue radiation dose constraints.

Organs at risk	Dose Constraints
Uninvolved liver	Mean dose <30 Gy
	$V_{30\ Gy}$ <30%*
Stomach and duodenum	D_{2cc} <45 Gy§
Spinal cord	Maximum point dose < 50 Gy
Kidney	Mean dose < 18 Gy
If mean kidney dose to one kidney >18 Gy, $V_{6\ Gy}$ (remaining kidney) <30%	

* VX Gy is defined as the percent of the liver volume that received x Gy or more. § DX cc is defined as the dose received by x cc of the organ volume.

© 2020 by the authors. Submitted for possible open access publication under the terms and conditions of the Creative Commons Attribution (CC BY) license (http://creativecommons.org/licenses/by/4.0/).
